# Fiber Intervention Study in Prader-Willi Syndrome: Insights into Metabolic and Microbiota Shifts

**DOI:** 10.1210/clinem/dgaf142

**Published:** 2025-03-01

**Authors:** Qiming Tan, Ye Peng, Edward C Deehan, Flavio T Vieira, Brian Wan Ping Ho, Shima Afhami, Eytan Wine, Karen L Madsen, Catherine J Field, Mohammadreza Pakseresht, Olga Ilkayeva, Christopher B Newgard, Jens Walter, Hein Min Tun, Andrea M Haqq

**Affiliations:** Department of Pediatrics, Faculty of Medicine and Dentistry, University of Alberta, Edmonton, AB, Canada T6G 2B7; Microbiota I-Center (MagIC), Hong Kong SAR, 999077, China; JC School of Public Health and Primary Care, Faculty of Medicine, The Chinese University of Hong Kong, Hong Kong SAR, 999077, China; Li Ka Shing Institute of Health Sciences, The Chinese University of Hong Kong, Hong Kong SAR, 999077, China; Department of Food Science and Technology, University of Nebraska, Lincoln, NE 68588, USA; Nebraska Food for Health Center, Food Science & Technology, Lincoln, NE 68588, USA; Department of Pediatrics, Faculty of Medicine and Dentistry, University of Alberta, Edmonton, AB, Canada T6G 2B7; Microbiota I-Center (MagIC), Hong Kong SAR, 999077, China; Cumming School of Medicine, University of Calgary, Calgary, AB, Canada T2N 1N4; Department of Pediatrics, Faculty of Medicine and Dentistry, University of Alberta, Edmonton, AB, Canada T6G 2B7; Department of Medicine, University of Alberta, Edmonton, AB, Canada T6G 2B7; Department of Agricultural, Food and Nutritional Science, University of Alberta, Edmonton, AB, Canada T6G 2P5; Department of Agricultural, Food and Nutritional Science, University of Alberta, Edmonton, AB, Canada T6G 2P5; Duke Molecular Physiology Institute and Sarah W. Stedman Nutrition and Metabolism Center, Duke University School of Medicine, Durham, NC 27710, USA; Department of Medicine, Division of Endocrinology, Metabolism, and Nutrition, Duke University School of Medicine, Durham, NC 27710, USA; Duke Molecular Physiology Institute and Sarah W. Stedman Nutrition and Metabolism Center, Duke University School of Medicine, Durham, NC 27710, USA; Department of Medicine, Division of Endocrinology, Metabolism, and Nutrition, Duke University School of Medicine, Durham, NC 27710, USA; Department of Pharmacology and Cancer Biology, Duke University Medical Center, Durham, NC 27710, USA; APC Microbiome Ireland, University College Cork, Cork T12 K8AF, County Cork, Ireland; School of Microbiology, University College Cork, Cork T12 K8AF, County Cork, Ireland; Department of Medicine, University College Cork, Cork T12 K8AF, County Cork, Ireland; Microbiota I-Center (MagIC), Hong Kong SAR, 999077, China; JC School of Public Health and Primary Care, Faculty of Medicine, The Chinese University of Hong Kong, Hong Kong SAR, 999077, China; Li Ka Shing Institute of Health Sciences, The Chinese University of Hong Kong, Hong Kong SAR, 999077, China; Department of Pediatrics, Faculty of Medicine and Dentistry, University of Alberta, Edmonton, AB, Canada T6G 2B7; Department of Agricultural, Food and Nutritional Science, University of Alberta, Edmonton, AB, Canada T6G 2P5

**Keywords:** Prader-Willi syndrome, hyperphagia, gut microbiota, dietary fiber, metabolic health

## Abstract

**Context:**

While increased fiber intake may benefit appetite and metabolism in the general population, its effects in individuals with Prader-Willi syndrome (PWS), a condition characterized by hyperphagia, obesity, and metabolic dysregulation, remain to be explored.

**Objective:**

This study assessed the effects of a fiber intervention on hyperphagia, metabolic health, and gut microbiota in individuals with PWS, and explored associations between changes in health markers and shifts in microbiota.

**Methods:**

Participants received either a high-dose fiber intervention (35 g/day) or a control for 3 weeks. Following a washout period of 4 to 8 weeks, participants switched treatments for another 3 weeks. Fecal (bacterial 16S ribosomal RNA) and blood (immunometabolic markers, targeted metabolomics) samples were collected before and after each treatment.

**Results:**

Fourteen participants (with a median age of 13.6 years, 8 [57.1%] were female) reported high tolerance to the fiber intervention. While it did not significantly alter hyperphagia or key metabolic markers, the fiber intervention led to shifts in gut microbiota diversity and increased the abundance of beneficial bacteria, such as *Bifidobacterium longum* and *Faecalibacterium prausnitzii*. Additionally, it altered fecal and serum metabolites, including a decrease in branched-chain fatty acids and an increase in serum C4-OH acylcarnitine.

**Conclusion:**

While this study did not observe significant changes in primary or secondary endpoints, it suggests that a short-term high-fiber intervention may induce beneficial shifts in gut microbiota and microbial metabolites in individuals with PWS. Further research is warranted to investigate the long-term effects and potential therapeutic applications of fiber interventions in PWS.

Prader-Willi syndrome (PWS) is a neurodevelopmental disorder that results from the lack of expression of maternally imprinted genes located in the paternal chromosome 15q11 to q13 region (1). Hyperphagia, a central feature of PWS, manifests as an intense and persistent hunger sensation, accompanied by a lack of normal satiety, an extreme drive to consume food, food preoccupation, and food-related behavior problems (2). Without proper management, individuals with PWS may develop severe obesity in early childhood (2). Hyperphagia remains a considerable clinical challenge, as current treatment modalities have demonstrated limited efficacy in its management (3). The severity of symptoms can vary, but hyperphagia typically requires a restricted lifestyle and constant supervision of food intake. This limitation impedes individuals with PWS from leading independent lives and adversely affects the quality of life both for patients and their caregivers (2). Effective therapeutics to attenuate hyperphagia is of top priority for caregivers and individuals with PWS (4).

The gut microbiota plays a crucial role in host health and has been implicated in various diseases, including PWS (5-7). Dietary fiber, a key modulator of the gut microbiome, offers a promising avenue for intervention. Fiber promotes satiety through mechanisms such as delayed gastric emptying, slowed nutrient absorption, and its fermentation by gut microbiota, which yields beneficial short-chain fatty acids (SCFAs). These SCFAs play a multifaceted role, serving as an energy source for colonocytes, regulating appetite signaling, mitigating inflammation, and optimizing glucose and lipid metabolism (8). While the relationship between fiber, satiety, and food intake is complex and not fully understood, some studies suggest that high-fiber interventions may contribute to increased satiety and reduced food intake in certain populations (9). Given the challenges of hyperphagia in PWS, and the established role of high-fiber interventions in promoting satiety, increasing fiber intake represents a potential dietary strategy to manage food intake in this population.

Previously, Zhang et al (5) demonstrated the positive effect of a diet rich in nondigestible carbohydrates on weight management and gut microbiota modulation in children with PWS, supporting the potential of dietary fiber interventions for this population. However, in their study, participants remained hospitalized throughout the study and traditional Chinese medicinal foods were used in the intervention, which limits the generalizability of their findings to individuals in North America, where dietary patterns and environmental exposures are distinct.

We hypothesized that a high-dose fiber intervention would induce a reduction in hyperphagia, as measured by the Hyperphagia Questionnaire for Clinical Trials (HQ-CT). Based on clinical expertise, a 20% reduction in HQ-CT scores is considered to be a clinically meaningful improvement. We also anticipated improvements in metabolic health outcomes and health-relevant changes in the gut microbiota and serum metabolome. To test this hypothesis, we used readily available fibers and assessed their effects on hyperphagia in children and adults with PWS in free-living conditions. Furthermore, we evaluated the effect of the fiber intervention on metabolic health outcomes and the fecal microbiota and determined whether physiological effects induced by the fiber intervention were associated with compositional and functional changes in fecal microbiota.

## Materials and Methods

### Participant Recruitment

Children and young adults aged 5 to 25 years were eligible to participate in the study if they had a confirmed diagnosis of PWS, free thyroxine concentrations within the normal range, and maintained a stable body weight (body mass index [BMI] percentile fluctuation <5% in the past 2 months). While this 2-month period does not represent long-term weight stability, it was chosen to exclude participants with active weight gain or loss, which could confound the interpretation of HQ-CT changes. This criterion balanced the need to control for recent weight fluctuations with the feasibility of recruiting a representative PWS sample. Participants were also required to have a stable growth hormone dose over the prior 6 months. Exclusion criteria included the presence of any other major medical condition (eg, diabetes mellitus, chronic inflammatory bowel disease, chronic severe liver disease, kidney disease, neurologic disorders), as well as the use of investigational drugs (within the past year), antibiotics (within the past 60 days), probiotics, and prebiotics (within the past 30 days). Participants were recruited from the Stollery Children's Hospital pediatric endocrine clinics in Edmonton, Alberta, Canada, as well as through PWS organizations in the United States and Canada between 2019 and 2022. Based on the prior PWS intervention study by Zhang et al (5), we anticipated a within-person SD of 4.5 in the hyperphagia score. A sample size of 20 participants would provide 80% power to detect a statistically significant difference in HQ-CT pre and post intervention with 95% CI, assuming a 20% change. All study procedures were performed at the University of Alberta. The study protocol was approved by the University of Alberta's Health Research Ethics Board (Pro00069477) and prospectively registered November 5, 2019, with ClinicalTrials.gov (NCT04150991). Written informed consent and assent were obtained from all participants and parents before participation.

### Study Design

**Figure 1.**
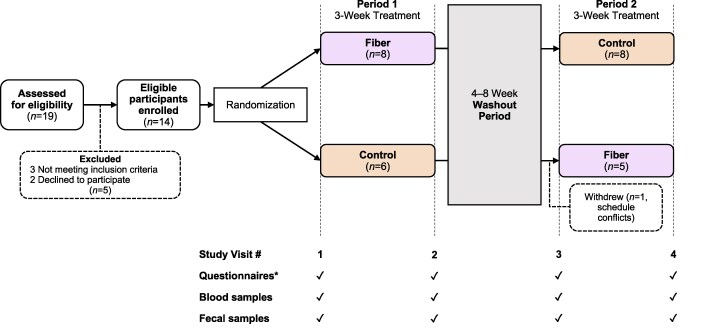
Study design of the crossover, randomized controlled trial. Participants were randomly assigned to either a fiber-first (35 g/day) or control-first sequence, receiving each treatment for 3 weeks, separated by a 4- to 8-week washout period. Outcomes were measured at 4 time points before and after each 3-week treatment period. *Questionnaires included 3-day dietary record, the Hyperphagia Questionnaire for Clinical Trials, gastrointestinal tolerability questionnaire, and visual analog scales for subjective sensations associated with eating.

This was a single-blind (participants), randomized, controlled, crossover, exploratory trial (Fig. 1). Participants were randomly assigned to either a fiber-first or control-first sequence, receiving each treatment (fiber [35 g/day] or control) for 3 weeks, separated by a 4- to 8-week washout period. A key aspect of this crossover study was the measurement of all outcomes at 4 time points before and after each 3-week treatment period. To capture these measurements, participants were asked to attend 4 study visits in total. All visits were conducted in the morning at the University of Alberta Hospital (Edmonton, Alberta, Canada) following an overnight 10-hour fast. At each visit, participants provided completed gastrointestinal (GI) tolerability questionnaires (maintained daily throughout each treatment period), 3-day dietary records (completed during the week prior to the visit), fecal and blood samples, and completed a subjective appetite questionnaire. Caregivers also completed the HQ-CT at each visit.

### Dietary Intervention

The fiber intervention included a powdered fiber mixture and a fiber-rich plant-based patty. To improve tolerability and adherence, a gradual dose escalation was implemented. Participants initially received 17.5 g/day of fiber for the first week, increasing to 35 g/day for the remaining 2 weeks of the intervention. The fiber mix was composed of acacia gum (TIC Gum Arabic; Ingredion), fructooligosaccharides (FOS; NUTRAFLORA*P*-95; Ingredion), and resistant maltodextrin (Fibersol; Archer Daniels Midland Company and Matsutani Chemical Industry Co). The structural diversity of these fibers may lead to distinct effects on the gut microbiota (Supplementary Table S1) (10, 11). The fiber patties were made from chickpeas, yellow split peas, black beans, and type 2 resistant starch (RS2; HI-MAIZE 260; Ingredion) (Supplementary Table S2) (11). The control treatment was prepared using an isocaloric amount of maltodextrin and patties made from white jasmine rice and cooked using the same method as the legume patties. To ensure that the organoleptic properties were similar between the fiber and control patties, sauces and seasonings were used to match the patties for color, taste, and texture. Three weeks' worth of doses were delivered in sealed opaque bags, each containing individually packaged, ready-to-use daily sachets of fiber mix or control. All patties were prepared in the Human Nutrition Research Unit metabolic kitchen and then individually packed in vacuum-sealed bags and stored frozen until consumption. Participants were advised to incorporate the fiber mix into their preferred foods and drinks daily, in addition to consuming one patty per day. To assess adherence to the treatment protocol, participants were instructed to return all provided sachets during their scheduled visits, and the unconsumed treatments were weighed and recorded. Participants were explicitly instructed to avoid prebiotics, probiotics, and antibiotics during the study period. Adherence to these restrictions was regularly monitored during follow-up visits to ensure compliance and minimize potential confounding effects on the gut microbiota. To further control for potential confounding, participants were asked to report any changes in their medication regimens, including gonadal steroid treatment, throughout the study.

### Dietary Intake

Participants were instructed to follow their usual eating pattern throughout the study. Participants with PWS, along with their caregivers, were required to complete a 3-day dietary record, including 2 nonconsecutive weekdays and 1 weekend day, before each visit. These records were then analyzed using Food Processor SQL (version 11.4, ESHA Research Inc).

### Hyperphagia

Hyperphagia symptoms were evaluated at each study visit using the HQ-CT (Supplementary Appendix A) (11). This 9-item validated questionnaire, with a total score range of 0 to 36, is designed for completion by caregivers of individuals with PWS and recognized as the consensus instrument for assessing hyperphagia in individuals with PWS in the context of clinical trials (12). Reductions in scores indicate improvements in hyperphagia. A subset of HQ-CT items (questions 1, 2, 5, 6, 8, and 9) are less influenced by environmental controls imposed by caregivers. HQ-CT question 9 (Q9) prompts caregivers to assess the extent of interference caused by hyperphagia in the daily life of individuals, providing an overall evaluation of improvement.

### Gastrointestinal Tolerability

To assess GI tolerability associated with the treatments, participants or their caregivers completed a daily questionnaire (Supplementary Appendix B (11)) assessing specific GI symptoms, using a 5-point severity scale (from “no symptom” to “severe”). We designed the study to allow flexibility in who completed the questionnaires, recognizing that the optimal reporter might vary depending on the individual participant and their circumstances. Participants who felt comfortable reporting on their GI symptoms directly were encouraged to do so. For participants who preferred assistance or for whom self-report might be less reliable, caregivers were able to provide support in completing the questionnaires. Mean weekly scores were calculated for each GI symptom.

Participants or their caregivers also tracked the frequency of bowel movements per week. Additionally, participants or their caregivers, as appropriate, reported on overall physical well-being and tolerance to the fiber and control treatments (on a 5-point scale from “very well tolerated” to “not tolerated”) and rated the feasibility and acceptability of incorporating extra powder and patties into their diets (on a 4-point scale from “not difficult” to “very difficult”).

### Subjective Sensations Associated With Eating

Visual analog scales (VAS) were used to measure subjective ratings of hunger, fullness, desire to eat, and prospective food consumption (Supplementary Appendix C) (13). The ratings of subjective appetite sensations reflect the participant's motivational drive to eat (13). The questionnaire consisted of 6 questions, from which a composite score was calculated.

### Appetite-regulating Hormones and Risk Markers of Metabolic Disease

Fasting blood samples were obtained for the analyses of appetite-regulating hormones and risk markers of metabolic disease related to glycemia, systemic inflammation, and lipidemia. In addition, at each visit, participants with PWS underwent an oral glucose tolerance test (OGTT), during which they ingested either 75 g (weight ≥ 40 kg) or 50 g (weight < 40 kg) of glucose, and blood samples for glucose and insulin were obtained at 0, 60, and 120 minutes. Serum and plasma samples were collected using separation tubes and EDTA whole-blood tubes, respectively. SST tubes were precoated with aprotinin and EDTA tubes were precoated with 4-(2-aminoethyl)benzene sulfonyl fluoride hydrochloride to prevent hormone degradation (BD Biosciences). Aliquots of plasma and serum were frozen and stored at −80 °C until analysis. Glucagon-like peptide-1 (GLP-1), peptide YY (PYY), leptin, and adiponectin were measured in inhibitor -treated plasma by electrochemiluminescence immunoassay (K151ACM-1 [RRID: AB_3665458] for GLP-1, PYY, and leptin, and K151R9K-1 [RRID: AB_3076200] for adiponectin, MesoScale Discovery; intra-assay coefficient of variation 4.6%, 4.4%, 8.4%, 5.7%, respectively). Glucose, insulin, high-sensitivity C-reactive protein (hs-CRP), alanine transaminase (ALT), aspartate transaminase (AST), total cholesterol, high-density lipoprotein cholesterol (HDL-C), and triglycerides were quantified in serum on a Roche Cobas c503 analyzer (Roche Diagnostics). Low-density lipoprotein cholesterol (LDL-C) was then calculated using the Friedewald equation (14). To quantify insulin resistance, the homeostatic model assessment of insulin resistance (HOMA-IR) was calculated using the following equation: fasting glucose (mmol/L) × fasting insulin (µIU/mL) /22.5 (15).The Matsuda index, a simple method for approximating whole-body insulin sensitivity from the OGTT, was calculated from plasma glucose (mg /dL) and insulin (mIU /L) concentrations in the fasting state and during OGTT (15). Additionally, to quantify the total increased blood glucose and insulin during an OGTT, the incremental area under the glucose and insulin OGTT curve was calculated using the linear trapezoidal method in GraphPad Prism.

### Targeted Serum Metabolomics

Trimethylamine *N*-oxide (TMAO), choline, and betaine were analyzed in serum by liquid chromatography with tandem mass spectrometry (LC-MS/MS) as previously described (16, 17). Amino acids and acylcarnitines were analyzed in serum using flow injection electrospray ionization MS/MS with isotope or pseudo-isotope dilution methods (18, 19). Ceramides and sphingomyelins were analyzed in serum as described by Merrill et al (20).

### Fecal Sample Collection, DNA Sequencing, and Microbiota Analysis

The method for fecal sample collection was described in a previous publication (7). Fecal samples were aliquoted and immediately stored at −80 °C until shipment to Microbiome Insights Inc for sequencing. Fecal homogenates were introduced into a MoBio PowerMag Soil DNA Isolation Bead Plate, and DNA extraction was conducted according to MoBio's instructions on a KingFisher robot (MoBio Laboratories). Bacterial 16S ribosomal RNA genes were polymerase chain reaction–amplified using dual-barcoded primers designed to target the V4 region (515F 5′-GTGCCAGCMGCCGCGGTAA-3′, and 806R 5′-GGACTACHVGGGTWTCTAAT-3′), following the protocol outlined by Kozich et al (21). Subsequently, amplicons were sequenced using an Illumina MiSeq platform with the 300-bp paired-end kit (v.3). All samples from this study were included in the same run, ensuring consistency and comparability across the data set. Fecal microbiota data were analyzed using the QIIME 2 pipeline (22), where sequencing reads were denoised by DADA2 to generate amplicon sequence variants (ASVs). Taxonomy of the ASVs were assigned using a combination of the SILVA database (SSU Ref NR 138.1, August 2020) and the broader NCBI BLAST database for comprehensive sequence comparison. Interindividual β-diversity indices (Bray-Curtis dissimilarity, weighted and unweighted Uni-Frac distance), α-diversity index (Chao1 index and Shannon diversity), and bacterial taxonomic compositions were determined based on 12 500 rarefied sequences per sample.

### Fecal Short-Chain Fatty Acid and Branched-Chain Fatty Acid Quantification

Analysis of fecal SCFAs was conducted as previously described with slight modifications (23). Gas chromatography (Thermo Trace 1310) with a flame ionization detector (FID), using a Thermo TG-WAXMS A GC column, was used to analyze the fecal SCFA profile. Total SCFAs (acetate, propionate, butyrate, and valerate) and branched-chain fatty acids (BCFAs: isobutyrate, isovalerate) were quantified. Relative proportions of each SCFA were calculated by dividing individual SCFA concentrations by the total SCFA concentration × 100 (24).

### Statistical Analyses

Participants who completed the study protocol were included in the final analyses. To assess the overall effect of fiber, data from individuals whose treatment sequence was assigned fiber-first (n = 8) and assigned fiber-second (n = 5) were combined and analyzed together as the “fiber treatment” (n = 13), and as the “control treatment” (n = 13). Missing clinical data were addressed using multiple imputation. Little's MCAR test supported the assumption of missing at random data. Multiple imputation was conducted with SAS Enterprise Guide 8.3, based on a multivariate normal distribution (MVN). To assess changes in GI symptoms during fiber and control treatments, Friedman's 2-way analysis of variance was employed. To evaluate the effects of dietary fiber intervention on health-related outcomes, we employed linear regression modeling. Change scores for each outcome measure were derived by subtracting baseline values (visit 1 and visit 3) from the corresponding values at the end of each treatment period (visit 2 and visit 4). These change scores served as the dependent variables in our models. The primary independent variable was a binary indicator representing treatment assignment (0 = control, 1 = fiber), enabling us to estimate the differential effect of the fiber treatment relative to the control treatment, which served as the reference group. Initially, univariate linear regressions were performed to examine the unadjusted associations between the fiber intervention and changes in each health-related marker (HQ-CT, perceived satiety and hunger, and biomarkers). Subsequently, we constructed multivariate linear regression models to assess the independent effect of the fiber intervention, controlling for potential confounding factors. Model 1 adjusted for age, sex, while model 2 additional incorporated Tanner stage, treatment sequence, genetic subtypes, and race as covariates. The analyses were performed using SAS 9.4 and SPSS Statistics (v29.0.1.0).

The analyses of fecal microbiota and metabolomics data were conducted on the R (v4.3.2, 2023-10-31) platform. The annotated microbial abundance data were imported to Phyloseq (v1.46) via Qiime2R (v0.99.6). Raw serum metabolomic data were imported via MetaboAnalystR (v4.0.0) and subsequently normalized via log transformation and Pareto scaling, with any zero or missing values replaced by half of the smallest positive values in the data set (25). A Euclidean distance matrix of the serum metabolome was computed by Vegan (v2.6-4) for a principal component analysis. Pairwise comparisons of overall compositions of the fecal microbiota (Bray-Curtis dissimilarity) and serum metabolome (Euclidean distance) between sample groups, preFiber, postFiber, preControl, and postControl were conducted using pairwiseAdonis2 (v0.4.1) (26). Changes from baseline to the end of 3 weeks of treatment in the fiber (preFiber vs postFiber) and control (preControl vs postControl) treatments in microbial features and serum and fecal metabolites were tested using signed Wilcoxon rank-sum tests. Correlations between continuous variables were calculated using Spearman correlation tests. *P* less than .05 was considered statistically significant. False discovery rate correction was conducted for multiple comparisons.

## Results

### Baseline Characteristics of Study Participants

The participants' demographic and clinical characteristics are summarized in Table 1. In total, 14 participants (median age of 13.6 years [interquartile range; IQR 9.6-17.5 years], predominantly White, median HQ-CT total score of 9 [IQR 5.5-11.3]), were consented and enrolled in the study. Eight of them were randomly assigned to the fiber-first sequence and 6 to the control-first sequence. Thirteen participants completed the study protocol. One participant, who had been assigned to receive the control treatment first, withdrew during the washout period due to schedule conflicts (see Fig. 1).

**Table 1.** Baseline characteristics of study participants

Characteristic	n = 14
Sex (female) No. (%)	8 (57.1)
Age at recruitment, y	13.6 (9.6-17.5)
Genetic subtype No. (%)	
Deletion	9 (64.3)
mUPD	4 (28.6)
Other	1 (7.1)
Tanner stage No. (%)	
1	3 (21.4)
3	1 (7.1)
4	4 (28.6)
5	6 (42.9)
Race, No. (%)	
White	11 (78.6)
Multiracial	2 (14.3)
Black	1 (7.1)
Not Hispanic or Latino, No. (%)	14 (100)
HQ-CT	9 (5.5-11.3)
On rHGH treatment at enrollment	10 (71.4)
Weight status, No. (%)	
Normal weight	5 (35.7)
Overweight	2 (14.3)
Obesity	7 (50)
Fasting glucose, mg/dL	82.8 (78.3-89.1)
Fasting insulin, mU/L	10.8 (9.0-19.3)
HOMA-IR	2.2 (1.7-4.0)
Matsuda index	3.5 (2.4-4.8)
Triglycerides, mmol/L	0.8 (0.6-1.1)
Total cholesterol, mmol/L	4.6 (4.4-5.0)
HDL-C, mmol/L	1.3 (1.1-1.5)
LDL-C, mmol/L	2.8 (2.6-3.3)
ALT, U/L	18.5 (16.0-27.3)
AST, U/L	24.5 (20.0-27.3)
hs-CRP, mg/L	0.9 (0.5-3.8)
GLP-1, pmol/L	12.2 (11.0-15.8)
PYY, pg/mL	69.9 (59.3-84.6)
Leptin, ng/mL	11.3 (6.4-16.8)
Adiponectin, μg/mL	13.6 (7.1-21.6)

Data are presented as median (interquartile range 25th percentile-75th percentile), except for categoric data shown as count (%). Biological sex, age, race, genetic subtype, and Tanner stage were determined on the date of informed consent. Data for clinical variables and HQ-CT were obtained at visit 1. Race and genetic subtype were reported by the participants. Tanner stage 1—prepuberty, Tanner stages 2 and 3—early puberty, stage 4—late puberty, and stage 5—adult-level maturity. The classification of weight status was determined based on the BMI percentile appropriate for age. HOMA-IR was calculated by using the following formula: fasting glucose (mg/dL) × fasting insulin (mU/L)/405. Matsuda index is calculated by using the following formula: 10 000/√glucose minute 0 × insulin minute 0) (mean glucose [OGTT]) × mean insulin OGTT).

Abbreviations: ALT, alanine transaminase; AST, aspartate transaminase; BCFA, branched-chain fatty acid; BMI, body mass index; GLP-1, glucagon-like peptide 1; HDL-C, high-density lipoprotein cholesterol; HOMA-IR, homeostatic model assessment of insulin resistance; HQ-CT, Hyperphagia Questionnaire for Clinical Trials; hs-CRP, high-sensitivity C-reactive protein; LDL-C, low-density lipoprotein cholesterol; mUPD, maternal uniparental disomy; OGTT, oral glucose tolerance test; PYY, peptide YY; rHGH, recombinant human growth hormone.

### Dietary Intake

Participants who completed the study (n = 13) demonstrated excellent adherence to the fiber and control treatments, averaging 93.9% and 97.3% for powders and 98.8% and 100% for patties, respectively. Daily dietary fiber intake significantly increased by 134% during the fiber intervention (from 19.6 [IQR 13.1-27.8] g/day at baseline to 44.0 [IQR 41.2-52.9] g/day; *P* = .001; Supplementary Table S3) (11). As shown in Supplementary Table S3, this increase in total dietary fiber intake could be directly attributable to the added fiber from the fiber mix and patties, as intake of dietary fiber from habitual diet remained stable. Total carbohydrate intake increased significantly from baseline in the fiber and control treatments (preFiber vs postFiber; *P =* .033; preControl vs postControl; *P* = .007). During the fiber treatment, this increase was primarily due to the carbohydrate content of the added fiber mix and patties. During the control treatment, the increase in total carbohydrate was due to the addition of maltodextrin and rice patties. No significant changes were observed in total energy intake or other dietary components.

### Gastrointestinal Tolerability

The powder and patties were deemed highly feasible and acceptable, with the majority of participants finding them very easy to integrate into their daily routines or reporting minor difficulties. Only one participant found them somewhat difficult to implement. Additionally, both treatments were generally well tolerated by the study participants. A majority of participants (59%) reported no symptoms, while 37% experienced only very minor GI symptoms. During the fiber treatment, Friedman's 2-way analysis of variance revealed no significant differences between weeks in reported scores for stomach aches and pains, abdominal distension or bloating, flatulence, overall physical well-being and tolerance, or bowel movement frequency. This suggests that the fiber dose increasing from 17.5 g during week 1 to 35.0 g during weeks 2 and 3 did not lead to changes in GI symptoms. Overall, participants with PWS tolerated the additional fibers well, despite the higher daily dose of 35 g.

### Hyperphagia

No significant associations were found between the fiber treatment and any of the hyperphagia scores (total hyperphagia score, subset score, or Q9 score) in either the univariate or multivariate analyses (Table 2 and Supplementary Table S4) (11). Sequence group had no effect on the HQ-CT total score. Neither recombinant human growth hormone use nor genetic subtypes, when considered in conjunction with the treatment, significantly altered the change in HQ-CT score. Notably, however, individual responses varied considerably. Four participants experienced clinically meaningful reductions (>20%) in HQ-CT total scores regardless of treatment assignment, while 3 others experienced this reduction specifically during the control treatment period. One participant, in particular, showed a substantial reduction (67%) exclusively during the fiber intervention. Similarly, no significant associations were found between the fiber treatment and any of the VAS questionnaire scores in either the univariate or multivariate analyses (see Table 2 and Supplementary Table S4) (11).

**Table 2.** Univariate regression analysis of fiber intervention effects on hyperphagia and other health markers

Variable	β coefficient	*P*	Lower 95% CI	Upper 95% CI
**Questionnaires**				
HQ-CT total score	1.93	.22	−1.21	5.08
HQ-CT subset score	1.25	.32	−1.26	3.75
HQ-CT Q9 score	0.20	.50	−0.41	0.82
VAS Q1: thoughts about food	6.10	.65	−21.62	33.82
VAS Q2: perceived hunger	4.66	.73	−22.78	32.10
VAS Q3: perceived satiety	14.53	.33	−15.83	44.88
VAS Q4: desire to eat	−2.63	.83	−26.90	21.63
VAS Q5: capacity to eat	6.72	.59	−18.35	31.79
VAS Q6: urge to eat	8.95	.55	−21.66	39.55
**Markers of cardiometabolic disease**				
Glucose iAUC	537.57	.51	−1134.80	2209.93
Insulin iAUC	−385.56	.83	−4120.38	3349.25
HOMA-IR	−0.14	.79	−1.22	0.94
Matsuda index	0.34	.49	−0.66	1.35
Triglycerides, mmol/L	−0.09	.41	−0.32	0.13
Total cholesterol, mmol/L	0.10	.55	−0.24	0.45
HDL-C, mmol/L	−0.01	.71	−0.09	0.06
LDL-C, mmol/L	0.15	.36	−0.19	0.49
Non-HDLC, mmol/L	0.11	.49	−0.21	0.43
ALT, U/L	−1.14	.68	−6.86	4.57
AST, U/L	0.98	.67	−3.61	5.57
hs-CRP, mg/L)	−0.30	.67	−1.70	1.11
GLP-1, pmol/L	0.78	.47	−1.42	2.98
PYY, pg/mL	12.00	.22	−7.58	31.58
Leptin, ng/mL	−1.03	.67	−5.87	3.81
Adiponectin, μg/mL	1.65	.21	−1.01	4.30
**Targeted metabolomics**				
Citrulline, µM	−1.9022	.**049**	−3.79	−0.01
C4-OH acylcarnitine, µM	0.0197	.**04**	0	0.04

Data were analyzed using univariate linear regression to examine the effect of the interventions on HQ-CT and other health-related markers. Regression coefficients (β), *P* values, and 95% CIs are provided. The control treatment served as the reference treatment, representing the absence of the active intervention. For each outcome variable, we calculated the change from baseline to the end of the respective treatment period (visit 2 for the first treatment period, visit 4 for the second treatment period). These change scores were then regressed on a binary indicator variable representing treatment assignment (0 = control, 1 = fiber). The regression coefficient (β) represents the mean difference in the change from baseline for the outcome variable between the fiber and control treatments. A positive coefficient indicates a greater increase (or a smaller decrease) in the outcome variable in the fiber treatment compared to the control treatment. A negative coefficient indicates a smaller increase (or a greater decrease) in the fiber treatment. The HQ-CT subset score was calculated as the sum of the scores from questions 1, 2, 5, 6, 8, and 9 in the HQ-CT. Data presented for participants who completed the study (n = 13). *P* less than .05 indicates a statistically significant difference. The use of bold font indicates values that are statistically significant.

Abbreviations: ALT, alanine transaminase; AST, aspartate transaminase; GLP-1, glucagon-like peptide-1; HQ-CT, Hyperphagia Questionnaire for Clinical Trials; HDL-C, high-density lipoprotein cholesterol; HOMA-IR, homeostatic model assessment of insulin resistance; hs-CRP, high-sensitivity C-reactive protein; iAUC, incremental area under the curve; LDL-C, low-density lipoprotein cholesterol; Non-HDLC, non–high-density lipoprotein cholesterol; PYY, peptide YY; VAS, visual analog scale.

### Metabolic and Inflammatory Markers

Univariate and multivariate analyses showed that the fiber treatment did not significantly change the assessed immunometabolic markers relative to the control treatment, which included GLP-1, PYY, leptin, adiponectin, OGTT glucose incremental area under the curve (iAUC), OGTT insulin iAUC, HOMA-IR, Matsuda index, triglycerides, total cholesterol, HDL-C, LDL-C, ALT, AST, or hs-CRP (see Table 2 and Supplementary Table S4) (11).

### Targeted Metabolomics

No significant changes were observed in the overall serum metabolome of participants throughout the study (Supplementary Fig. S1) (11). The univariable regression analysis revealed a statistically significant direct effect of the fiber treatment on change in serum concentrations of citrulline (β = −1.90; *P* < .05; 95% CI, −3.79 to −0.01) and C4-OH acylcarnitine (β = .02; *P* = .04; 95% CI, 0-0.04) (see Table 2). For C4-OH, the significance of association remained after adjusting for multiple covariates (β = .02; *P* = .04; 95% CI, 0-0.04) (see Supplementary Table S4) (11). Additionally, multivariate analysis revealed a significant association between the fiber treatment and change in C4/Ci4 (β = −.03; *P* = .04; 95% CI, −0.06 to 0). There was no significant association between the fiber treatment and changes in serum TMAO concentrations or any other measured metabolites (see Supplementary Table S4) (11).

### Fecal Microbiota Composition

**Figure 2.**
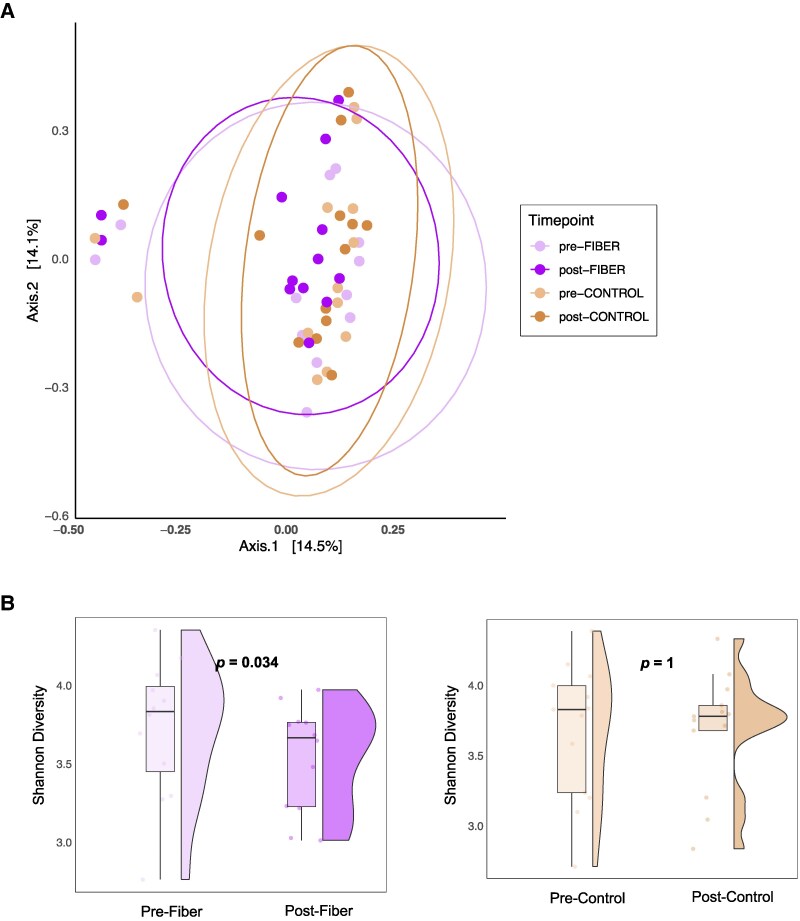
Gut microbiota diversity at different time points. A, Bray-Curtis dissimilarity. Each data point represents an individual participant, reflecting shifts in their gut microbial community composition. Bray-Curtis distance was calculated for each pair of samples. These distance matrices were then analyzed using software tools (Phyloseq and QIIME2) for transformation, ordination, and statistical testing with pairwise Adonis2 (v0.4.1). B, Shannon diversity index. This index measures both the richness (number of species) and evenness (relative abundance of species) within the gut microbiota. Higher values indicate greater diversity.

A total of 1 853 889 raw reads were obtained with an average of 25 130 reads per sample (range, 12 456-45 457 reads). To visualize microbiota composition similarity, Bray Curtis, Unweighted Unifrac, and Weighted Unifrac distances were calculated, followed by Principal Coordinates Analysis (PCoA) ordination. Samples collected at baseline before either treatment showed no significant difference between the fiber and control treatments (preFiber vs preControl). However, after the treatment, the community composition of the fiber treatment was significantly different from that of the control treatment (Fig. 2A, Bray-Curtis, postFiber vs postControl; *P* = .01). No significant differences were found in other β-diversity metrics among different time points (Supplementary Fig. S2) (11). Additionally, participants on the fiber treatment exhibited lower α-diversity (Shannon index; preFiber vs postFiber; *P* = .03) in the posttreatment total bacterial community (Fig. 2B). No changes in α-diversity of the total bacterial community were detected in the participants on the control treatment (preControl vs postControl).

**Figure 3.**
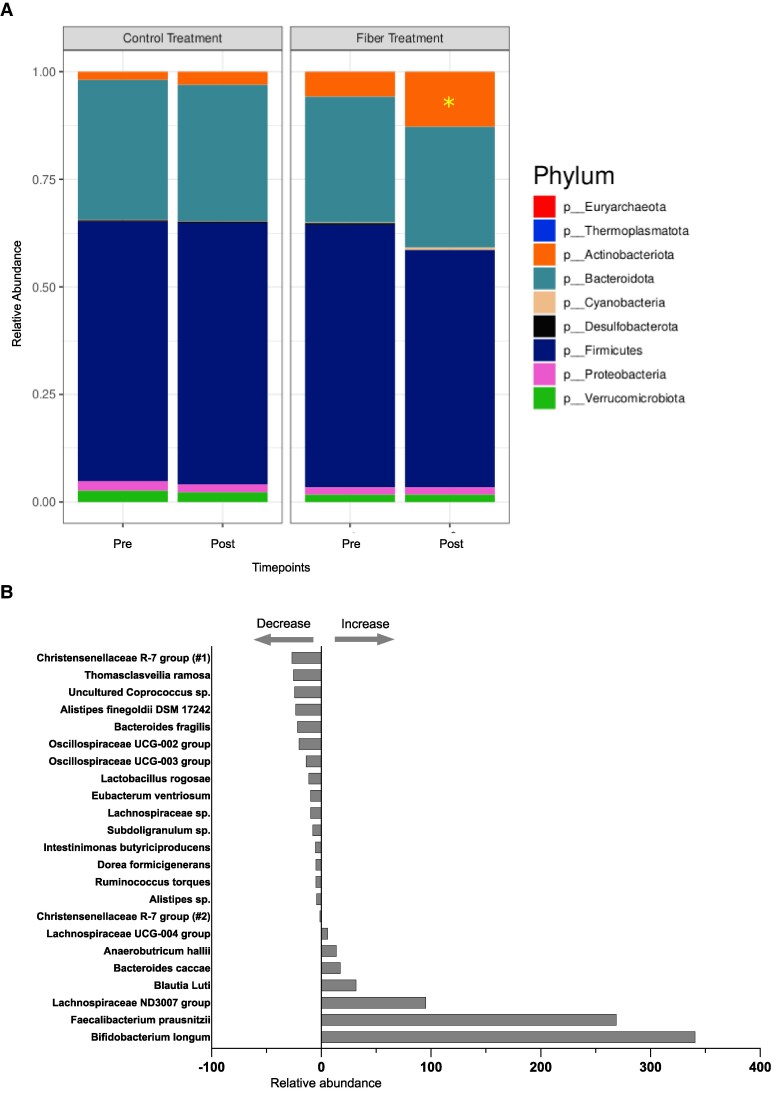
Effect of fiber intervention on gut microbiota composition. A, Phylum-level shifts. Relative abundances of major bacterial phyla are compared before and after fiber or control interventions. Within-subject changes over time were assessed using Wilcoxon matched-pairs signed-rank tests (*P < .05 indicates statistical significance). B, Significant amplicon sequence variant (ASV) changes with fiber. This panel highlights specific bacterial strains (ASVs) that showed significant changes in relative abundance after the fiber intervention, but not in the control group. Bars represent the median change in abundance, and ASVs are sorted by the magnitude of this change. Full details on preintervention and postintervention abundances, as well as individual changes for all ASVs, can be found in Supplementary Table S5 (11).

Fig. 3A shows changes in the gut microbiota at the phylum level for the fiber and control treatments at baseline and post treatment, and the relative abundances of genus-level gut microbiota composition at these time points is presented in Supplementary Fig. S3 (11). While the relative abundance of the most abundant bacterial phyla remained largely stable within individuals across time points, there was a notable increase in the relative abundance of *Actinobacteriota* after the fiber treatment (Fig. 3A; preFiber vs postFiber; *P* = .01; change in relative abundance in fiber vs control treatment; *P* = .03). We further analyzed changes in the relative abundance of ASV after the fiber intervention. This analysis revealed 23 ASVs changed significantly after the fiber treatment relative to baseline (preFiber vs postFiber), with 7 increasing and 16 decreasing (Fig. 3B and Supplementary Table S5) (11). Specifically, there was a substantial increase in the relative abundance of putatively beneficial taxa, including ASVs related to *Bifidobacterium longum* (median increase of 341%; IQR, 100.25%-876.25%; *P* = .02), *Faecalibacterium prausnitzii* (median increase of 269%; IQR, 127%-437.5%; *P* = .03), *Lachnospiraceae* ND3007 group (median increase of 95.5%; IQR, 19.25%-174.25%; *P* < .001), *Blautia luti* (median increase of 32%; IQR, 2%-97.25%; *P* = .04), *Bacteroides caccae* (median increase of 17.5%; IQR, 0%-38%; *P* = .02), *Anaerobutricum hallii* (median increase of 14%; IQR, 1.5%-23%; *P* = .03), and *Lachnospiraceae* UCG-004 group (median increase of 6%; IQR, 0%-18.25%; *P* = .04). Conversely, taxa related to *Christensenellaceae* R-7 group (median decrease of 27%; IQR, −105.5% to 0%; *P* = .02) and *Thomasclavellia ramosa* (median decrease of 25.5%; IQR, −54% to −0.75%; *P* = .04) showed the most pronounced decreases. We also observed significant reductions in several taxa with complex associations to gut health, including *Alistipes finegoldii* and *Bacteroides fragilis*, both of which have been linked to inflammation in certain contexts. Additionally, the decrease in *Ruminococcus torques*, a keystone degrader of intestinal mucin glycoprotein, may have implications for gut metabolic function and warrants further investigation.

### Fecal Microbial Functions

**Figure 4.**
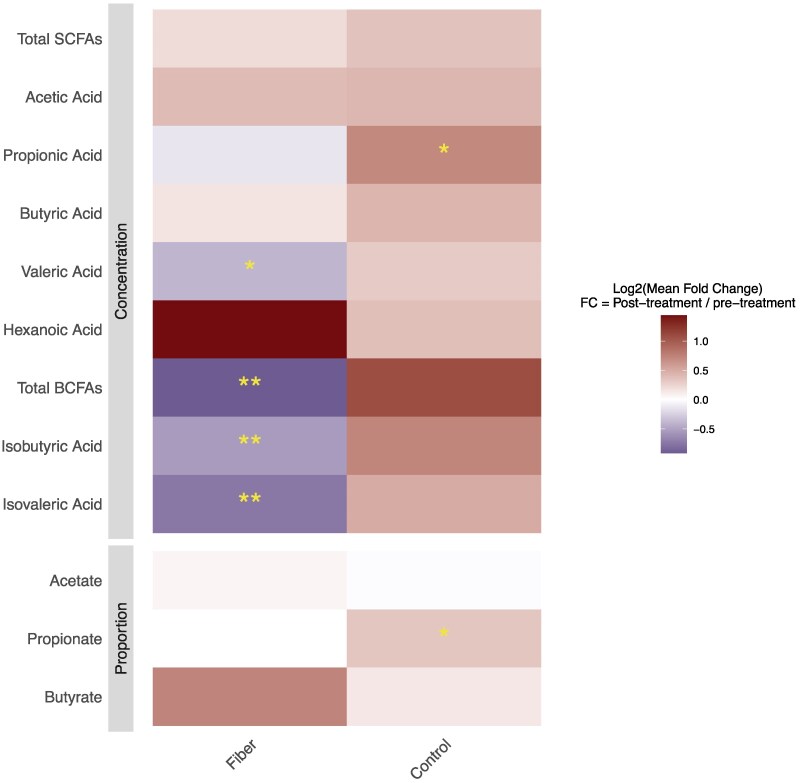
Changes in fecal short-chain fatty acid (SCFA) and branched-chain fatty acid (BCFA) concentrations following treatment. Concentrations in the fiber and control groups were compared by calculating log2 mean fold change (posttreatment/pretreatment values). Invalid fold changes (eg, division by zero) were excluded. Log2 transformation was applied to the mean estimate, and differences were assessed using a Wilcoxon matched-pairs signed-rank test. *P* less than .05 indicates a statistically significant difference. *P less than .05; **P less than .01.

To investigate the effect of treatments on the functional profile of the fecal microbiota, we measured fecal concentrations of SCFAs and BCFAs. The fiber treatment did not exhibit a statistically significant change in total SCFA levels compared to baseline (preFiber vs postFiber), nor were there significant differences in total SCFA levels observed between the fiber and control treatment (postFiber vs postControl) (Fig. 4). Furthermore, apart from valeric acids (*P* = .03), individual SCFAs or their relative proportions did not change significantly after the fiber intervention (preFiber vs postFiber). However, significant reductions compared to baseline were observed in total fecal BCFAs (*P* = .0002), as well as the individual BCFAs isobutyric (*P* = .03) and isovaleric acids (*P* = .0005), after the fiber treatment (preFiber vs postFiber), suggesting a reduction in the microbial fermentation of branched chain amino acids. In contrast, participants demonstrated a significant increase (preControl vs postControl) in fecal propionic acid concentration after the control treatment (*P* = .04). This unexpected increase could be attributed to the starch retrogradation that occurs in the control patties during cooking and cooling, leading to a higher content of resistant starches that are selectively fermented by gut microbes to propionic acid.

### Associations Between Shifts in Fecal Bacteria and Clinical Indices

Correlation analysis revealed significant associations between specific fecal metabolites and bacterial taxa (Supplementary Fig. S4) (11). C4/Ci4 was positively associated with *R torques* (ρ = 0.65; *P* = .02) but negatively associated with unclassified *Lachnospiraceae* (UCG-003) (ρ = −0.58; *P* = .05), unclassified *Ruminococcaceae* (UCG-002) (ρ = −0.60; *P* = .04), *Christensenellaceae* R-7 group (ρ = −0.54; *P* = .07), and Proteobacteria (ρ = −0.52; *P* = .09). C4-OH was positively associated with *B luti* (ρ = 0.71; *P* = .01) and negatively associated with UCG-003 (ρ = −0.52; *P* = .08). Citrulline was negatively associated with *B luti* (ρ = −0.51; *P* = .09) and positively associated with *F prausnitzii* (ρ = −0.49; *P* = .02).

## Discussion

In this study, we investigated the effects of a 3-week, high-dose, diverse fermentable fiber intervention on hyperphagia, metabolic health outcomes, and fecal microbiota in individuals with PWS. While the fiber intervention did not significantly alter the primary outcome (change in HQ-CT score), we observed notable changes both in the serum metabolomic profile and fecal bacterial community. These changes included shifts in diversity of the total bacterial community and fecal concentrations of BCFAs, as well as serum citrulline and C4-OH acylcarnitine.

Considerable variation was observed in participants' responses to the fiber intervention across behavioral, clinical, and gut microbiota measures. This heterogeneity may be due to factors such as disruption of structured routines, common in PWS, which can trigger anxiety, stress, and increased food-seeking. The degree of sensitivity to routine disruption and adaptability to dietary changes varies among individuals with PWS, which may contribute to the observed variation in HQ-CT total scores. Our study's free-living design may also have contributed to response variability. Finally, the 90-day study by Zhang et al (5), which reported improvements in various health markers, suggests that extended interventions may be necessary to allow for physiological adaptations and broader health benefits to emerge.

Metabolomic profiling provides insights into dietary effects on metabolism. A previous study in children with PWS demonstrated that a low-carbohydrate, high-fat diet led to increased plasma concentrations of free fatty acids and most acylcarnitines (including C4-OH) compared to a low-fat, high-carbohydrate diet (27). Our study observed significant alterations in C4-OH and C4/Ci4 with the fiber intervention, suggesting that dietary changes can modify certain acylcarnitines. Monitoring these changes could offer a personalized approach to assessing metabolic responses to dietary interventions in PWS. Acylcarnitines are primarily derived from fatty acid oxidation (FAO) but can also be formed from amino acids and glucose metabolism (28). A recent study has shown that adults with PWS and obesity exhibit increased levels of medium- and short-chain acylcarnitines, suggesting incomplete oxidation of fatty acids in the later steps of the process. This impaired FAO may be linked to reduced skeletal muscle mass and altered branched-chain amino acid catabolism in PWS (29).In our study, the observed changes in C4-OH and C4/Ci4 could indicate activation of either FAO or branched-chain amino acid catabolism, or both, potentially increasing ammonia production. We also found that citrulline, involved in ammonia detoxification, decreased with the fiber intervention, possibly due to increased utilization in the urea cycle (30). We observed specific gut microbes correlating with serum C4-OH, C4/Ci4, and citrulline, suggesting these microbes may influence the production or regulation of these metabolites and play a role in the metabolic response to dietary fiber. Further research is needed to elucidate the mechanistic link between dietary fiber, gut microbes, and serum metabolite, as well as the clinical implications of these fiber-induced changes.

Our study revealed significant postfiber intervention shifts in gut bacteria composition, including increases in potentially beneficial taxa such as *Blongum* and *F prausnitzii*. These changes align with previous findings demonstrating the prebiotic potential of the fibers used in our intervention (31-34). Concurrently, we observed changes in overall community composition (β-diversity) and a reduction in α-diversity. This reduction in diversity aligns with prior studies using short-term dietary modifications and might be attributed to the targeted effects of specific fibers on certain bacteria and the competitive dynamics within the gut microbial ecosystem (35). The introduction of novel fiber sources and potential displacement of less-adapted taxa could have contributed to decreased richness and evenness (35). However, as highlighted by Zhang et al (5), understanding functional changes in the microbiome may be more crucial than focusing solely on diversity when evaluating the effect on host health. Further investigations using metagenomic analysis or functional assays could elucidate how these shifts in bacterial populations translate to changes in the gut microbiota's overall metabolic capacity.

While total SCFAs remained unchanged following fiber intervention compared to control, we observed a significant reduction in BCFAs. This decrease in BCFAs suggests a potential reduction in protein fermentation during the fiber intervention. Fecal BCFA levels have been proposed as markers of protein fermentation, a process that can lead to the production of harmful byproducts (eg, ammonia, phenol, *p*-cresol, or biogenic amines) with potential implications for colonic epithelial health (36). However, there are also studies suggesting potential roles of BCFAs in regulating glucose and lipid metabolism (37, 38). The overall effect of BCFAs likely depends on production levels, gut microbial composition, and individual health status, warranting further investigation. These results, alongside the observed changes in gut microbiota, highlight the potential for fiber interventions to modulate gut microbial activity and its downstream metabolic products in individuals with PWS.

Our study offers several key strengths that adds valuable knowledge to the underexplored field of gut microbiota modulation in PWS. The crossover design strengthens internal validity, and our comprehensive assessment of both the microbiota and metabolome offers a multidimensional perspective. The intervention itself employed a high daily dose of diverse fiber sources. This approach is more likely to capture the multifaceted effects of dietary fiber on the gut microbiota, as compared to studies using limited fiber types or lower dosages. However, our study has limitations that warrant consideration. The short intervention period may not fully capture the long-term effects of dietary fiber shifts on the gut microbiota and metabolic health. Additionally, the relatively small sample size might limit the generalizability of our findings to a broader PWS population. It also prevents us from exploring potential differences in fiber response among individuals with distinct genetic subtypes within the PWS population. Based on the small sample size and limited corrections for multiple comparisons, our findings should be interpreted cautiously. Nonetheless, our findings provide guidance for future research on PWS with larger sample sizes. Moreover, the HQ-CT, while a valuable tool, is known to have limitations in its ability to detect subtle changes or capture the full spectrum of hyperphagic behaviors, particularly in individuals living in highly structured food environments (12). Future research could benefit from incorporating additional behavioral or physiological measures to provide a more comprehensive assessment.

To conclude, while a 3-week fiber intervention in individuals with PWS did not induce statistically significant changes in hyperphagia or immunometabolic markers, our study provides valuable insight to inform the use of dietary fibers for this population. It is important to acknowledge that the effects on hyperphagia in free-living individuals with PWS may be fiber dependent, subtle, and vary considerably between individuals. We observed notable shifts in the serum metabolome and gut microbiota, revealing intriguing associations between specific metabolites and microbes that warrant further exploration. These findings highlight the potential for personalized fiber interventions tailored to individual gut microbiota profiles and metabolic responses. Building on these results, future research should explore dose-response relationships and the effects of individualized fiber interventions to fully unlock the therapeutic potential of dietary fiber for individuals with PWS, ultimately leading to improved health outcomes and quality of life.

## Data Availability

The amplicon sequencing data have been deposited in the European Bioinformatics Institute (EBI) under accession number PRJEB77518. The SCFA and serum-targeted metabolomic data are available as a draft on Figshare, with the reserved digital object identifier (DOI) https://doi.org/10.6084/m9.figshare.26246132. Remaining deidentified individual participant data, methods, and study materials will be made available from the corresponding authors on reasonable request.

## Clinical Trial Information

ClinicalTrials.gov identifier number NCT04150991 (registered <2019-11-05>).

## Abbreviations

ALT: alanine transaminase
AST: aspartate transaminase
ASV: amplicon sequence variant
BCFA: branched-chain fatty acid
BMI: body mass index
FAO: fatty acid oxidation
GI: gastrointestinal
GLP-1: glucagon-like peptide-1
HQ-CT: Hyperphagia Questionnaire for Clinical Trials
HDL-C: high-density lipoprotein cholesterol
HOMA-IR: homeostatic model assessment of insulin resistance
hs-CRP: high-sensitivity C-reactive protein
iAUC: incremental area under the curve
LC-MS/MS: liquid chromatography with tandem mass spectrometry
LDL-C: low-density lipoprotein cholesterol
Non-HDLC: non–high-density lipoprotein cholesterol
OGTT: oral glucose tolerance test
PWS: Prader-Willi syndrome
PYY: peptide YY
SCFA: short-chain fatty acid
TMAO: trimethylamine *N*-oxide
VAS: visual analog scale
